# Effects of Paratuberculosis Vaccination at Different Ages in a Dairy Goat Herd: A 2-Year Follow-Up

**DOI:** 10.3390/ani12223135

**Published:** 2022-11-14

**Authors:** Miguel Fernández, Marcos Royo, Miguel Fuertes, Noive Arteche-Villasol, M. Carmen Ferreras, Julio Benavides, Valentín Pérez

**Affiliations:** 1 Departamento de Sanidad Animal, Facultad de Veterinaria, Campus de Vegazana, Universidad de León, 24007 León, Spain; 2 Departamento de Sanidad Animal, Instituto de Ganadería de Montaña (CSIC-ULE), 24346 Grulleros, Spain

**Keywords:** goats, ruminants, dairy, paratuberculosis, vaccination, pathology, *Mycobacterium avium* subsp. *paratuberculosis*, heterologous protection, immune response

## Abstract

**Simple Summary:**

Paratuberculosis is an economically important disease due to its negative effect on animal production. One of the tools for controlling this disease is vaccination. The peripheral humoral and cellular immune responses and animal losses—regardless of reason—depending on whether or not they were vaccinated and their age, were studied for almost two years in a herd of dairy goats. The immune response was greater and more persistent when vaccination took place at an earlier age. Moreover, animals vaccinated at this time showed heterologous protection, as evidenced by a decrease in the number of dead and culled animals compared to those not vaccinated. Based on the cellular immune response, no cross-reactivity was observed when an intradermal skin test for tuberculosis diagnosis was employed.

**Abstract:**

Vaccination could be considered as an effective method for paratuberculosis control, although controversial, with a need for investigation in some aspects. The objective of this study was to evaluate the effect of vaccination, depending on the age of the animals, on their immune response, the reduction of paratuberculosis cases, mortality and culled animals in a commercial dairy herd. Goats from three different ages were immunized with the inactivated Gudair^®^ vaccine. Peripheral antibody and IFN-γ output were evaluated for 21 months post-vaccination (mpv) and intradermal skin tests (IDSTs) for tuberculosis, with avian- and bovine-purified protein derivatives (PPD), were carried out at 6 and at 18 mpv to evaluate the humoral and cellular immune peripheral responses, respectively. The number of dead or culled animals, regardless of the reason, was also monitored and the causes of death determined by pathological examination. A significant increase in the production of IFN-γ was observed in all the vaccinated groups when the blood samples were stimulated with avian PPD, from 3 mpv to 18 mpv, and with bovine PPD, between 3 and 21 mpv. Moreover, serum antibody levels increased between 3 and 21 mpv in all vaccinated groups. The highest levels were found in animals vaccinated at 5 months, and the lowest in adult individuals. No positive reactants to tuberculosis were found by intradermal skin test. No animal losses associated with clinical paratuberculosis were detected in any of the groups. The number of total culled animals was significantly lower in the vaccinated than in the unvaccinated groups, especially on 1.5-month-old vaccinated kids. These results suggest that vaccination of paratuberculosis, especially in young animals, could induce heterologous protection.

## 1. Introduction

Paratuberculosis or Johne’s disease is a widespread chronic infectious disease of ruminants, caused by *Mycobacterium avium* subsp. *paratuberculosis* (MAP), that has been associated with substantial economic losses due to a decrease in production and losses of animals. Although different strategies have been proposed, vaccination could be an effective method for controlling paratuberculosis, since it decreases fecal shedding of MAP and achieves a reduction in clinical cases [1,2,3,4,5,6,7,8]. In fact, MAP-infected animals are considered a real concern in animal husbandry due to their capability to intermittently shed the viable pathogen into the environment [3,4]. However, the use of vaccination has been hampered mainly due to the interference with the eradication programs based on immunological testing and elimination of animals identified as infected and can interfere with the interpretation of intradermal skin tests (IDSTs) for bovine tuberculosis [9].

Some studies of vaccination in young animals about one month of age [7,8] and other studies in adults [7,10] have shown protective effects, but no consensus has yet been reached regarding the best time for immunization. The most logical age of vaccination, to avoid transmission of MAP, would be in the first days of life [11]. However, the fact that current paratuberculosis vaccines induce an immune response and an intense and severe local reaction at the inoculation point [12,13], but do not prevent infection, has led to their use in adult animals [2,10,14]. In this sense, there is a previous study comparing different ages of vaccination, where peripheral immune response was more intense and longer in animals that were vaccinated at higher age in relation to a better maturation of the immune system [15], but the real efficacy of vaccination against paratuberculosis was not evaluated in that work.

Thus, this study was performed in a commercial herd where paratuberculosis was previously confirmed by the demonstration of clinical cases and positive serology, with the main aim of assessing the effect of the age of vaccination on the control of the disease, by evaluating the peripheral immune response and the number of losses or early discarding of animals.

## 2. Materials and Methods

### 2.1. Ethical Information

Experimental procedures with animals that constitute this study received a positive evaluation from the animal experimentation committee at the University of León, with authorization reference OEBA-ULE-016-2017, and ethics committee CEE-IGM2017-001.

### 2.2. Animals and Experimental Design

This study was performed at an intensive dairy goat farm, with a census of approximately 800 adult animals of Murciano-Granadina breed, located in Aguilar de Campos (Valladolid, Spain). This herd had tuberculosis-free status, since it was subjected to official eradication programs and no positive reactors had appeared for the last five years. The farm also had a history of clinical cases of paratuberculosis during at least the previous five years, confirmed by the existence of lesions in animals showing clinical signs and serology. These animals were necropsied and a severe granulomatous enteritis with the presence of high numbers of acid-fast bacilli in the tissues and fecal swabs, consistent with paratuberculosis, was diagnosed. In addition, serological examination by indirect ELISA test was carried out at different periods, in a total of 87 animals, with a seroprevalence around 1% of examined individuals.

Goats were intensively managed in recently built barns, with feeding adapted to the production stage and rearing only own females without animal introduction from outside. Annual health programs included deworming and vaccination against clostridiosis and contagious agalactia. To date, paratuberculosis vaccination has never been performed.

In this study, a total of 190 female goats from different ages according to the productive stratum were included. The age of the experimental groups was decided on the basis of the regular management of the herd: newborn kids, replacement or adult animals. A total of 77 does between 1.5 and 5 years, 79 doelings of approximately 5 months and 34 kids around 1.5 months of age were employed. Half of them from each group were vaccinated (V) and the remaining animals were not vaccinated (NV). Thus, animals were classified in six groups: 39 adult females vaccinated (AdultsV), 38 adult females not vaccinated (AdultsNV), 40 goats of 5 months vaccinated (5 MV), 39 goats of 5 months not vaccinated (5 MNV), 19 1.5-month-old vaccinated kid goats (1.5 MV) and 15 1.5-month-old non vaccinated kid goats (1.5 MNV). Goats were vaccinated with the commercial vaccine Gudair^®^ (Vetia Animal Health, S.A., Madrid, Spain), composed of the 316 strain of MAP inactivated by heat, with mineral oil as adjuvant. One milliliter of vaccine was subcutaneously inoculated, according to manufacturer instructions, in the post-scapular area of the back. Control animals, kept non-injected, were managed together with the same goats from the herd under the same conditions.

### 2.3. Clinical Follow-Up and Necropsies

During the 21 months after vaccination an evaluation of the number of losses was carried out. With the objective of determining the cause of death, and the presence or absence of gross and microscopic lesions associated to MAP infection, all dead or culled animals in the herd, regardless of reason for death, were collected and brought to the Veterinary Faculty of León, where complete necropsy was carried out. After recording all gross lesions observed, appropriate tissue samples were taken, always including ileocecal valve, mesenteric lymph node and different sections from the ileum and the jejunum with and without Peyer’s patches. All were fixed in 10% buffered formalin and routinely processed for histological examination. Diagnosis of the main causes of death was performed on the basis of the main gross and histological lesions showed by the animals [16]. For the identification and classification of lesions related to MAP infection, guidelines proposed in previous studies were used [17].

### 2.4. Peripheral Immune Response Evaluation

**Figure 1:**
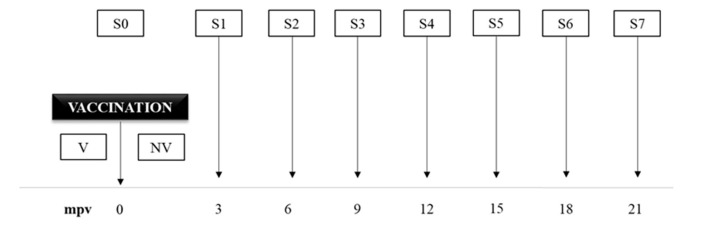
Schedule of the study design and blood sampling times (S0 to S7). S: sampling; mpv: months post vaccination. V: vaccinated; NV: non-vaccinated.

A total of 8 blood samples from each goat of the six groups were collected every three months (S0 to S7) (Figure 1). S0 sampling was performed just before vaccine administration. Blood samples were obtained from the jugular vein, using 10 mL Vacutainer^®^ both heparinized and without heparin tubes (Becton Dickinson, Plymouth, UK). After collection within 4 h and under refrigeration, samples were moved to the laboratory to start the different procedures.

Peripheral immune response was evaluated by cellular and humoral immune response tests: the cellular immune response was assessed by interferon gamma (IFN-γ) release assay (IGRA) and comparative IDST; the humoral response through an indirect ELISA test for antibody detection. IGRA was performed following previous studies [18] where whole blood samples were stimulated with avian and bovine purified protein derivative (PPD) (CZ Veterinaria, Porriño, Spain) in a final concentration of 30 µL/ml before IFN-γ determination in the plasma obtained after centrifugation, using a commercial test, following the manufacturer’s instructions (“BOVIGAM^®^, Thermo Fisher Scientific, Waltham, MA, USA). Results were expressed as a quotient between the mean of the optical density (O.D.) of the avian PPD-stimulated plasma and the mean of O.D. of the same plasma incubated with PBS.

A comparative IDST was performed at 6 mpv and 18 mpv in all animals. The antigens employed were avian and bovine PPD (CZ Veterinaria, Porriño, Pontevedra, Spain). Before procedure, skin fold thickness was measured in the neck area. Skin test was performed by intradermal administration of 0.1 mL of avian PPD and bovine PPD on the right side of the lower part of the neck. Both inoculations were separated approximately by 13 cm [9]. The immunological response against these antigens was evaluated by measuring the skin fold thickness of these inoculation sites after 72 h.

According to the regional animal health authorities, comparative IDST was considered positive for tuberculosis diagnosis, when the skin-fold thickness of the bovine PPD inoculation point was 4 mm higher than the inoculation site of avian PPD. An animal was considered suspicious when the increase in skin-fold thickness at the bovine PPD inoculation site was between 2 and 4 mm than the reaction at the site of the avian PPD inoculation. Comparative IDST was considered negative when the increase in the skin-fold thickness at the bovine inoculation point was lower than avian inoculation site.

Evaluation of antibodies against MAP was evaluated by indirect ELISA test (ELISA ID Screen^®^ Paratuberculosis Indirect, IDvet, Grabels, France), following the manufacturer instructions. The results were expressed as a percentage of increase of the mean O.D. of each tested sample in relation the mean O.D. of the positive control.

### 2.5. Statistical Analysis

Data on antibody and IFN-γ production were subjected to analysis of variance using the GLM (General Linear Model) procedure of the SAS statistical package (SAS Institute Inc., Cary, NC, USA). The results of the O.D. indexes obtained in the indirect ELISA and IFN-γ tests were logarithmically transformed to make them suitable for analysis of variance. Differences among the experimental groups at each time of sampling were evaluated using the Student’s *t*-test for pair-wise comparisons and the Tukey–Kramer correction for multiple comparisons. Frequency data associated with the number of losses in each experimental group was analyzed by chi-squared analysis and Fisher exact test (if some expected results from chi-squared test were lower than 5). In all the cases, statistic signification level was 0.05%.

## 3. Results

### 3.1. Evaluation of the Number of Losses

During the study, there were 19 total losses (10% of the experimental animals), distributed as follows (Table 1): 11 goats were lost during the first year (5.79%) and 8 in the second year (4.47%). During the study, losses from all non-vaccinated groups (n = 13) were significantly higher (*p* < 0.05) than losses from vaccinated groups (n = 6). In animals vaccinated at 5 months of age, the same number of deaths were found in each group. However, significant differences (*p* < 0.05) were observed in the 1.5-month-old kids (1.5 M) between vaccinated (1.5 MV) and not vaccinated (1.5 MNV) groups during the first year. In the second year, there was no loss in any of the 1.5-month groups, vaccinated (1.5 MV) or not vaccinated (1.5 MNV). In the rest of the groups, differences were not significant.

Causes of losses were variable. The three kids from the 1.5 MNV group died during the first four months after vaccination due to a severe and acute fibrinonecrotic pneumonia, affecting both lungs, associated with *Mannheimia haemolytica* infection. Among 5 M groups, two animals from the 5 MV group died during the first year due to clostridiosis and hemorrhagic septicemia, respectively, and another one from the 5 MNV group also showed lesions consistent with clostridiosis. During the second year, one goat from 5 MV showed lesions consistent with pregnancy toxemia, and another two (group 5 MNV) exhibited clostridiosis and cerebrocortical necrosis. In the Adult groups, among the animals lost during the first year, three were discarded due to acute staphylococcal mastitis (one vaccinated and two non-vaccinated), one died due to mechanical asphyxia (vaccinated) and the remaining one was culled due to reproductive failure. During the second year, one vaccinated goat was discarded due to acute mastitis, and the remaining four animals showed lesions consistent with acute mastitis (two goats), pregnancy toxemia or severe necrotic endometritis after parturition.

**Figure 2:**
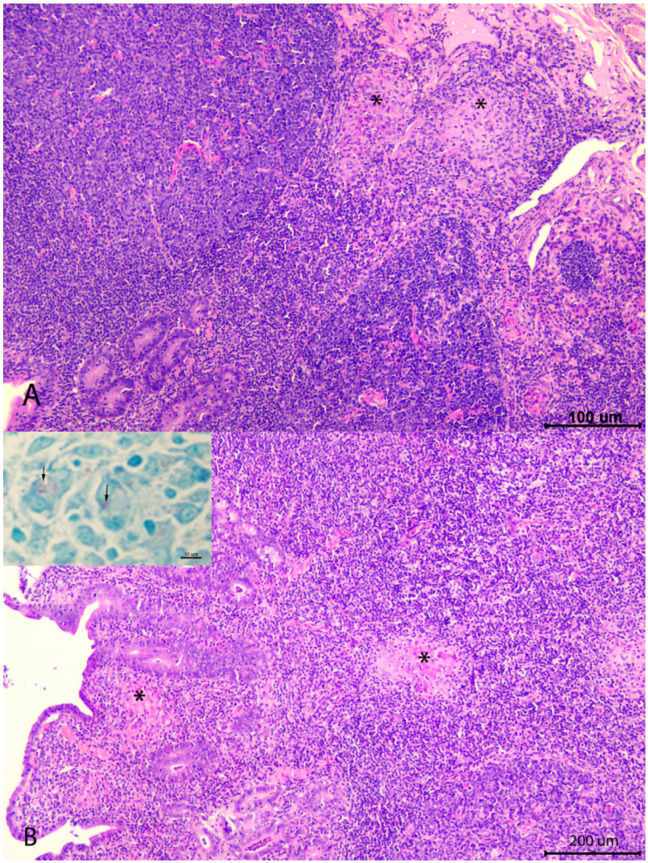
Granulomatous lesions associated with paratuberculosis infection found in the necropsied goats. (**A**): focal lesion, formed by small granulomas (*) located exclusively in the interfollicular area of the Peyer’s patches. Hematoxylin-eosin (HE) (**B**): multifocal lesion, composed of granulomas (*) located both in the interfollicular area on the Peyer’s patches and related lamina propria. HE. Inset: small numbers of acid-fast bacilli (arrows) seen in the lesion located in the lamina propria. Ziehl–Neelsen stain (inset).

Paratuberculosis was not identified as a cause of death in any case. Only subclinical microscopic lesions were observed in three adult goats (Figure 2). Focal forms, characterized by small granulomas exclusively located in the jejunal lymphoid tissue, were seen in two goats, from the AdultsV and NV groups, which died due to acute mastitis and asphyxia. One goat from the AdultsNV group showed a multifocal lesion, with granulomas located in the lymphoid tissue and also in the lamina propria related to Peyer’s patches, but not as severe as to cause a diffuse enteritis related to overt clinical signs, where a low number of acid-fast bacilli was detected. This animal was culled due to reproductive failure.

### 3.2. Cellular Immune Response Evaluation

**Figure 3:**
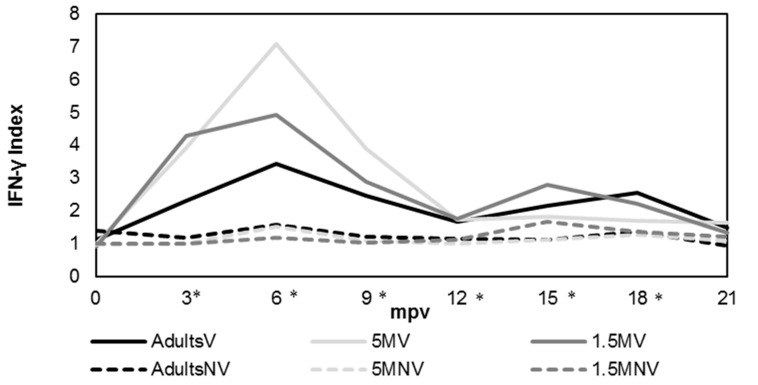
Evolution in the INF-γ production after stimulation of blood with avian PPD according to each experimental group. AdultsV: vaccinated at more than 1.5 years; AdultsNV: not vaccinated at more than 1.5 years; 5 MV: vaccinated at 5 months; 5 MNV: not vaccinated at 5 months; 1.5 MV: vaccinated at 1.5 months; 1.5 MNV: not vaccinated at 1.5 months; mpv: months post vaccination; * significant differences (*p* < 0.05) among vaccinated and not vaccinated groups. Results were expressed as a quotient between the mean O.D. of the avian PPD-stimulated plasma and the mean O.D. of the same plasma incubated with PBS.

Figure 3 shows the evolution of IFN-γ production after stimulation of blood with avian PPD over 21 months after vaccination (eight samplings) by IGRA assay. The obtained values were expressed as the mean value shown by the animals from each experimental group. Kinetics of production were similar in the three vaccinated groups. Significant differences (*p* < 0.05) were observed between vaccinated and not vaccinated groups from 3 mpv to 18 mpv at the moment these differences disappeared. The maximum level of production of this cytokine was obtained at 6 mpv in all vaccinated groups, and was significantly (*p* < 0.05) different from not vaccinated groups.

A large variation between the animals was observed along the experiment. The statistical analysis performed showed that the highest levels of IFN-γ production were seen between 6 and 9 mpv in the case of the group vaccinated at 5 months. These levels were significantly (*p* < 0.05) different from other vaccinated groups and the differences disappeared at 9 mpv. Also in this group, the highest differences were observed between vaccinated and unvaccinated goats, from 3 mpv to 12 mpv (*p* < 0.001) and 15 mpv (*p* < 0.05). On the other hand, the production of IFN-γ from 1.5 MV animals did not show significant differences with 5 MV at 3 mpv, but there were differences between 6 and 9 mpv (*p* < 0.05). The AdultsV group presented lower levels of this cytokine than 1.5 MV group at 3 mpv (*p* < 0.05) and with 5 MV from 6 to 9 mpv (*p* < 0.05). Later, differences were not observed.

**Figure 4:**
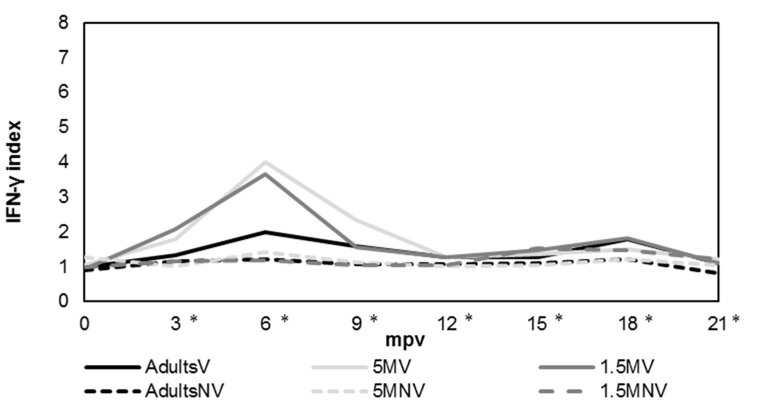
Evolution in the INF-γ production after stimulation of blood with bovine PPD according to each experimental group. AdultsV: vaccinated at more than 1.5 years; AdultsNV: not vaccinated at more than 1.5 years; 5 MV: vaccinated at 5 months; 5 MNV: not vaccinated at 5 months; 1.5 MV: vaccinated at 1.5 months; 1.5 MNV: not vaccinated at 1.5 months; mpv: months post vaccination; * significant differences (*p* < 0.05) among vaccinated and not vaccinated groups. Results were expressed as a quotient between the mean O.D. of the bovine PPD-stimulated plasma and the mean O.D. of the same plasma incubated with PBS.

When samples were stimulated with bovine PPD (Figure 4), the IFN-γ production followed a similar pattern as when incubated with avian PPD, but always with values significantly lower (*p* < 0.05) in vaccinated groups over the duration of the study. As for avian PPD, there was a wide variation in the values of this cytokine production between the animals during the experiment. According to the statistical analysis performed, in all vaccinated groups, the levels of this cytokine were significantly (*p* < 0.05) higher than those of not vaccinated groups between 3 and 21 mpv. According to age of vaccination, 5 MV group showed the highest production, with significant differences (*p* < 0.05) with AdultsV group between 3 and 9 mpv, and with 1.5 MV group at 6 mpv. Between 1.5 MV and AdultsV groups, there were only significant differences (*p* < 0.05) in samplings performed at 3 and 9 mpv.

A comparative IDST test was carried out in two periods, at 6 and 18 mpv. No animal showed a positive result for tuberculosis diagnosis in any of those times. In vaccinated animals, the increase of the fold skin thickness in the inoculation site of bovine PPD was always lower than the increase in thickness of the inoculation area of avian PPD. Considering single IDST values, no animal showed a positive results either.

### 3.3. Humoral Immune Response

**Figure 5:**
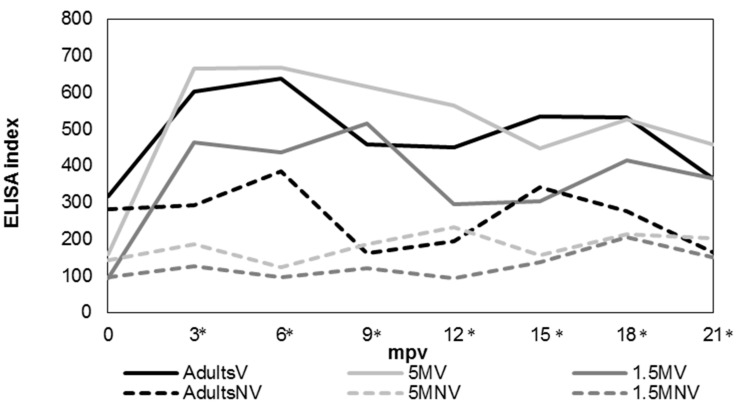
Evolution of antibodies production against MAP according to each experimental group. AdultsV: vaccinated at more than 1.5 years; AdultsNV: not vaccinated at more than 1.5 years; 5 MV: vaccinated at 5 months; 5 MNV: not vaccinated at 5 months; 1.5 MV: vaccinated at 1.5 months; 1.5 MNV: not vaccinated at 1.5 months; mpv: months post vaccination; * significant differences (*p* < 0.05) among vaccinated and not vaccinated groups.

The humoral immune response was assessed by an indirect ELISA test (Figure 5). The obtained values were expressed as the mean value showed by the animals from each experimental group. Antibody levels from vaccinated groups were significantly higher (*p* < 0.05) than in not vaccinated groups between 3 mpv and 21 mpv. However, among vaccinated groups, antibody levels were higher in 5 MV, with significant differences (*p* < 0.05) with the 1.5 MV group between 3–6 mpv and 12–15 mpv and with the AdultsV group between 9–12 mpv (*p* < 0.05).

## 4. Discussion

There is a scarcity of publications where vaccination against paratuberculosis under field conditions in caprine species is evaluated. Thus, this study was designed to investigate the peripheral immune response induced by vaccination, and its effect on the herd disease status, according to different ages of vaccination. The use of inactivated vaccines had already demonstrated efficacy in reducing the number of clinical cases, as well as in MAP shedding [4,5,8,10,19].

In this study, no clinical cases of paratuberculosis were recorded over the limited time of the study in any of the groups, vaccinated or unvaccinated, and thus, no effect of vaccination in disease control could be inferred. This could probably be due to the fact that this herd showed a low grade of prevalence of MAP infection, as the low rates of serological response identified in the previous screenings could suggest, supporting the fact that the effect of paratuberculosis vaccine on this infection would depend on the status of the herd in which it is implemented [13]. Adult animals, vaccinated or not, were the only ones in which paratuberculosis lesions were detected, and only microscopically. The only animal that showed the most advanced lesion (multifocal) was an unvaccinated goat. This fact, despite the low number of cases, could be an indicator of the beneficial effect of paratuberculosis vaccine on the control of the disease, limiting the progression of the lesions to focal forms observed in vaccinated goats, while in those not vaccinated, infection might progress to more severe forms [2,7,20]. Moreover, the existence of animals with focal lesions that could persist during their entire productive lives without being vaccinated has been pointed out [15,20,21,22].

An interesting result of this study was the positive effect that vaccination showed over the general reduction in the number of losses in the herd, for any cause, during the study (21 mpv). In vaccinated groups, 6.12% of losses were incurred while not vaccinated groups lost a 14.13% of the animals and, as stated, paratuberculosis was not among the reasons of death. In a previous 4-year follow-up carried out in goats vaccinated at 1 month of age, a 38% reduction in losses from vaccinated animals compared to animals without vaccination was found—although this difference was not significant, and the causes of death were not investigated [8]—that the authors associate with a decrease in the number of clinical cases of paratuberculosis. In our study, however, there were no clinical cases of paratuberculosis in any of the two groups (vaccinated or not vaccinated), but a reduction in the number of deaths was observed among vaccinated animals. Although the variety of etiologies involved and the low number of animals included in the study have to be considered, it is tempting to hypothesize that paratuberculosis vaccination could have had an influence.

Although a general reduction was observed, the 1.5 M group was the only one where significant differences in relation to vaccination status were observed, with no deaths recorded among vaccinated kids. It was noticeable that all the animals suffered from acute pneumonia that occurred during the first 4 months after vaccination. In humans, with tuberculosis vaccination (BCG), heterologous protection has been reported in children that was more intense in the three months after vaccination, decreasing with time, until one year post-vaccination [23]. A reduction in the general mortality unrelated to tuberculosis was observed in children vaccinated with BCG [23,24,25,26]. Specifically, a significant decrease in the cases of bacterial pneumonia among vaccinated children (less than one year) was reported [27]. These results are in full agreement with the results observed in our study, where 1.5-month vaccinated goats (1.5 MV) presented an evident reduction in mortality, and all the losses in 1.5 MNV group were due to acute bacterial pneumonia too. An explanation, in the case of tuberculosis, was that vaccination with BCG would induce a trained immune response [23,24,25,26,27,28], which combines an induction of innate immune response together T cell-mediated immune response. This synergic response must be produced by an epigenetic reprogramming of monocytes and a bigger production of cytokines such as epidermal growth factor (EGF), IL-6 or platelet-derived growth factor (PDGF) [29]. Evidence of this kind of response with the use of the BCG vaccine has been provided [23,24,25], but not in vaccination against paratuberculosis, although similar results to those observed in this study were previously reported in paratuberculosis-vaccinated cattle [7].

The fact that a trained immune response exists has been highlighted in humans when children were vaccinated with BCG [23,25,26]. In agreement with this result, in this study, the only group with significant differences was the one vaccinated at 1.5 months, raising the hypothesis that this response would occur predominantly in young animals with an immature immune system. All these findings support the possibility that a similar mechanism of trained immune response could exist in paratuberculosis vaccination, and efforts to elucidate their nature should be devoted in future studies.

On the other hand, this study has shown that vaccination induces an intense peripheral immune response, both cellular and humoral, measured by IGRA and indirect ELISA, respectively, as previous studies have documented in goats [8,10] and other ruminant species [7,30]. At 3 mpv, a significant increase in the production of IFN-γ after stimulation with avian PPD was observed, that lasted until 18 mpv, and the highest level was reached at 6 mpv. This result would be in agreement with other studies where the highest level appeared at 8.5 mpv (first sampling) [8] and differences disappeared at 23 mpv. However, in another study that employed the same vaccine in goats, differences lasted only until 9 mpv [9]. The only difference between both studies was the breed of the goats, Alpine versus Murciano-Granadina. In other species, the duration of the response was 7.5 mpv in lambs [30] and between 9 and 30 mpv in calves [12]. In our study, both responses were detected at 3 mpv, as in previous works [30,31]. Regarding the antibody levels, differences were seen until the end of the experiment between vaccinated and unvaccinated animals, in agreement with that observed previously [8].

In relation to paratuberculosis diagnosis in vaccinated animals, the results indicated that the vaccine interfered with immunological tests, either IGRA or indirect ELISA, at least until 18 or 21 mpv, respectively. Similar results have been also reported in other studies [8,32]. Regarding the relation between immune response and the protective effect of the vaccine, the cellular immune response was considered effective against MAP infection [7,33,34,35], so these animals should be protected for at least 2 years after vaccination. However, there are several studies that pointed out that protection offered by the vaccine would be longer, at least over the entire productive life of animals [10,19,36,37,38], so this capacity would not be related directly with the existence of high peripheral IFN-γ levels produced by sensitized blood lymphocytes. It has been reported that the effect of vaccination is the induction of a modification of the local inflammatory response, at intestinal level, able to control the progression of infection at this site [7,20]. This fact should not be related to peripheral high levels of IFN-γ since the existence of animals with focal lesions, associated with latent forms, in which the individual is able to control the progression of lesions that show negative IGRA responses, has been commonly found [21,22,39,40]. In addition, in cases of vaccination against tuberculosis there is controversy about whether the levels and duration of IFN-γ after vaccination are indicative of protection against mycobacteria [24]. In relation to the high antibody levels, the inoculation of the vaccine results in the presence of high numbers of mycobacteria in the vaccination nodule, and it is known that the antibody response in paratuberculosis is closely related to the presence of high numbers of bacteria in tissue [41,42,43,44]. Traditionally, this immune response has not been associated to protection against MAP infection, as opposed to cellular response [33,35], although a possible protective role of antibodies has been pointed out [45].

Another objective of this study was to investigate the influence of the age of animals at the time of vaccination over the induced immune response. For considering the results, it has to be taken into account that remarkable individual variations in the levels of IFN-γ production were observed during the experiment, and although differences were significant after statistical analysis in some samplings, these differences would probably be more accurately considered as tendencies. Thus, goats from the 5 MV group were those with the more intense peripheral immune responses, particularly the cellular one, between 6 and 9 mpv. These results agree with a previous study also carried out on goats [15]. Next, the 1.5 MV group showed the second-best response, and the lowest was detected in the AdultsV group. A higher grade of maturation of the immune system in animals at 5 months than at 1.5 months would be a possible explanation for these results [15], but this hypothesis does not explain the lower levels found in adult goats. Similar results have been found with the BCG vaccine, and previous immunizations with environment mycobacteria, or even in our case MAP, which was present in the herd, have been suggested as a possible explanation for being the cause of blocking or masking immune mechanisms in adult animals [46]. These mechanisms could be so intense that a great immune response would be mitigated, even though the more mature immune system [46].

Keeping in mind these results and considering that an increase in the cellular immune response would be associated to protective response of the vaccine, the most recommendable age of vaccination would be around 5 months. In our study, we could not confirm that this procedure is associated with a better protective effect, since no cases of paratuberculosis were detected in any group. Previous studies have demonstrated that a vaccine is effective if it is administrated in animals around 1 month of age, with the mission of obtaining protection before the first challenges with MAP in experimental and in field studies [8,30,46]. However, good results have been also achieved by vaccinating adult animals, presumably already infected [2,10], where vaccination could have a therapeutic effect. Although there are no studies that compare efficacy according to time in caprine species, in ovine species, vaccination between 3–8 months of age is recommended [47]. Although the paratuberculosis vaccine seems to be effective at any age, future field or experimental studies focused on the protective effect of the vaccine according to the age of vaccination would be of interest.

One of the disadvantages of vaccination against paratuberculosis is the cross reaction with the diagnosis of tuberculosis by immunological methods [9,48,49,50]. In the present study, vaccination induced the production of IFN-γ after bovine PPD stimulation, as observed previously [8,12]. Additionally, in the work by Mercier et al., 2014 [8], during the first 15.5 mpv, the levels of IFN-γ induced by both PPD were similar or even slightly higher in the case of bovine PPD. However, in our study, interferences between vaccination against MAP and tuberculosis diagnosis tests were not produced in any case, neither by comparative IDST nor single IDST. In another study performed on goats in France [32], the skin fold thickness in the inoculation area of bovine PPD was evaluated at 8 mpv and the mean was 3 mm, although some animals reached 10 mm, and 23% of the goats were positive by single IDST, although none were detected using comparative IDST. Perhaps the number of animals controlled, which was higher in this study than in our case, could explain these differences. In any case, these results indicate that only a low number of vaccinated goats would show interference with the official tuberculosis eradication campaigns, as previously seen in a study at a large scale that shows that this interference clearly diminishes with the time [51].

## 5. Conclusions

Vaccination against paratuberculosis could exert a heterologous protection in a dairy goat herd with clinical history of the disease, since the number of culled animals, regardless the cause, was significantly lower among vaccinated than in non-vaccinated animals, over a period of 21 months. This effect was more evident in kids vaccinated at 1.5 months and suggests that a mechanism of trained innate immune response, as previously seen in BCG vaccination, could also operate in paratuberculosis. Further studies involving more animals will be necessary in the future for the confirmation of this hypotheses. On the other hand, vaccination induces strong cellular and humoral peripheral immune responses, already detected at 3 mpv, that persist for almost 2 years. This response is influenced by the age at which the animals are vaccinated, being more intense and durable when vaccination takes place at 5 months, while lower and shorter in adult goats. In this study, no positive reactor to tuberculosis was found between vaccinated goats, neither by comparative IDST nor single IDST, suggesting that interference of tuberculosis diagnosis with paratuberculosis vaccination was reduced.

## Figures and Tables

**Table 1:** Losses of goats vaccinated with Gudair^®^ (V) or not vaccinated (NV) with percentages and distribution according to each experimental group throughout the period studied (1st and 2nd year, and total). AdultsV: vaccinated at more than 1.5 years; AdultsNV: not vaccinated at more than 1.5 years; 5 MV: vaccinated at 5 months; 5 MNV: not vaccinated at 5 months; 1.5 MV: vaccinated at 1.5 months; 1.5 MNV: not vaccinated at 1.5 months.

Groups	Number of Goats at Beginning	Number of Losses at Year 1 (%)	Number of Animals at Year 2	Number of Losses at Year 2 (%)	Total Number of Losses (%)
**AdultsV**	39	2 (5.13)	37	1 (2.70)	3 (7.69)
**AdultsNV**	38	3 (7.89)	35	4 (11.43)	7 (18.42)
**5 MV**	40	2 (5.00)	38	1 (2.63)	3 (7.50)
**5 MNV**	39	1 (2.56)	38	2 (5.26)	3 (7.69)
**1.5 MV**	19	0 (0.00)	19	0 (0.00)	0 (0.00)
**1.5 MNV**	15	3 (20.00)	12	0 (0.00)	3 (20.00)
**Total**	190	11 (5.79)	179	8 (4.47)	19 (19.00)

## Data Availability

Not applicable.
